# Malignant perivascular epithelioid cell tumor of the lung synchronous with a primary adenocarcinoma: one case report and review of the literature

**DOI:** 10.1186/s12885-019-5383-0

**Published:** 2019-03-15

**Authors:** Jikai Zhao, Haohua Teng, Ruiying Zhao, Wenjie Ding, Keke Yu, Lei Zhu, Jie Zhang, Yuchen Han

**Affiliations:** 1 Department of Pathology, Shanghai Chest Hospital, Shanghai Jiao Tong University, No. 241 West Huaihai Road, Shanghai, 200030 China; 2 Department of Bio-Bank, Shanghai Chest Hospital, Shanghai Jiao Tong University, Shanghai, China

**Keywords:** Perivascular epithelioid cell tumor, PEComa, Malignant, Clear cell tumor, Lung, Adenocarcinoma, Diagnostic criteria

## Abstract

**Background:**

Perivascular Epithelioid Cell Tumors (PEComa) is an extraordinarily rare mesenchymal neoplasm especially the malignant type originating from the lung. To date, only 8 cases of malignant or malignant potential pulmonary PEComa had been documented. Firm diagnostic criteria for malignant pulmonary PEComa need urgently to be established.

**Case presentation:**

We report a challenging case of malignant pulmonary PEComa combined with a primary adenocarcinoma in a 54-year-old man. The PEComa-like tumor showed strong Melan-A and weak transcription factor E3 (TFE3) protein expression but no TFE3 gene rearrangement. The carcinoma-like nodule was recognized as a poorly differentiated primary lung adenocarcinoma.

**Discussion and conclusions:**

Our case report was the first case of malignant pulmonary PEComa synchronous with a primary adenocarcinoma and studied the dilemma of diagnosing benign versus malignant criteria for this uncommon tumor.

## Background

Perivascular Epithelioid Cell Tumor (PEComa) is a rare mesenchymal neoplasm which is thought to originate from perivascular epithelioid cells showing both melanocytic and myogenic differentiation [1]. Although this tumor was first described as a benign lung tumor in 1971 by Liebow and Castleman, it is extraordinary rare especially the malignant type originating from the lung [2].

Most PEComas are thought to follow a benign behavior. Clear cell tumor, angiomyolipoma, lymphangioleiomyomatosis, sugar tumor of the lung are all synonyms for this morphologically and immuno-phenotypically similar lesions [3]. Though malignant PEComa had been described and studied at numerous sites including soft tissue [4] and gynecologic Origin [5], retroperitoneum [6], gastrointestinal [7] and uterine [8, 9], the diagnostic criteria and treatment strategy of malignant pulmonary PEComa have not been firmly established.

To date, only 8 cases of malignant or malignant potential pulmonary PEComa had been documented. We herein report a case of a 54-year-old man who synchronously presented with a well-demarcated mass in the right middle lobe and a small solid nodule in the left lower lobe. Since our case not only had a PEComa-like nodule and showed another solid nodule, the pressure on surgical management and frozen pathologic diagnosis is to judge whether the two tumors are homologous or heterogenous. The next challenge is to find and give preliminary evidence of histological features that determining its malignant behavior. Therefore, we described the difficulties of diagnosing malignant pulmonary PEComa in frozen and routine paraffin sections, and the predicament of surgical management we confronted. In this article, clinical and pathologic features were evaluated, immunohistochemical analysis and molecular alteration was studied.

## Case presentation

**Fig. 1:**
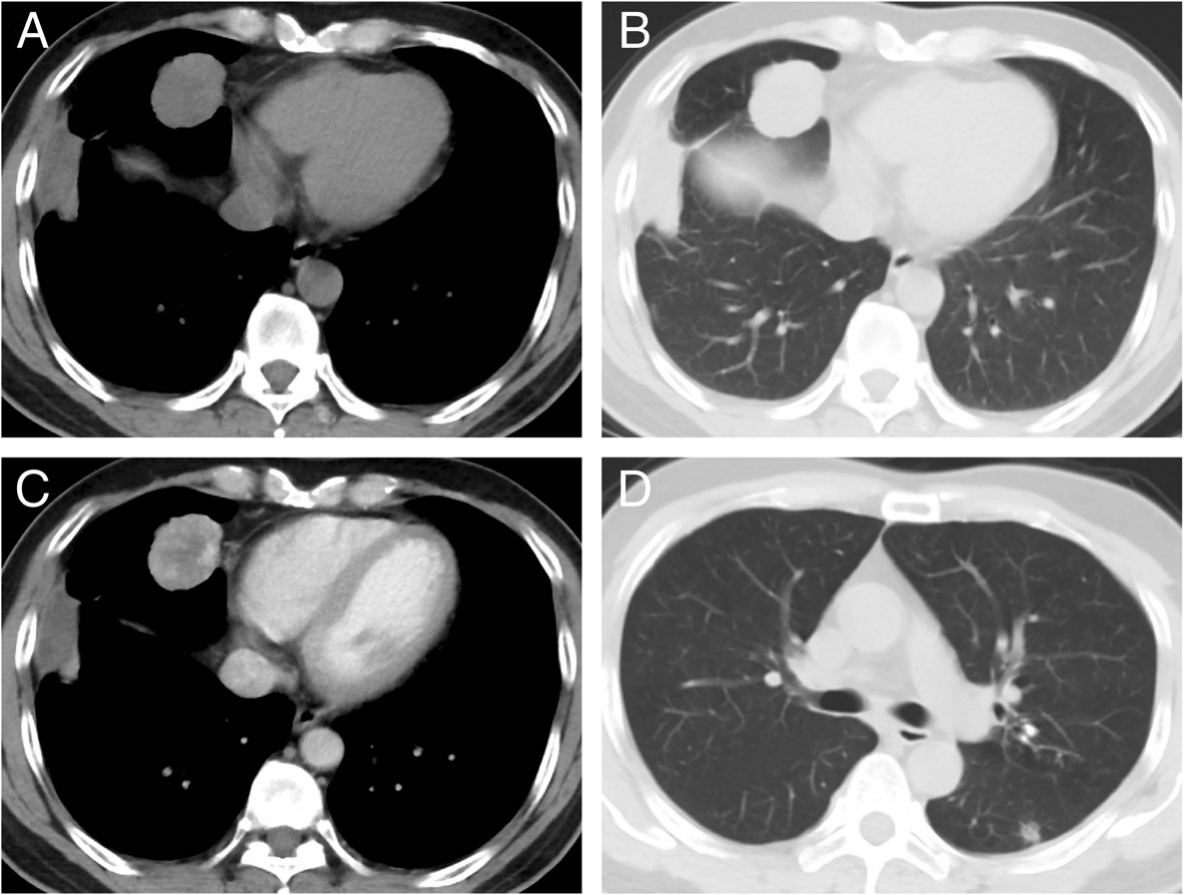
Computed tomography (CT) scan of the lung tumor. **a**, **b** A larger lesion was located in the right middle lobe with smooth boundary measured 4 cm × 3.5 cm × 2 cm, accompanied by heterogeneous enhancement (**c**). **d** Another small nodule about 11 mm was located in the left lower lobe with lobulated shape and spicules of margin

A 54-year-old man was admitted to Shanghai Chest Hospital due to pulmonary shadow incidentally detected on routine chest roentgenograms. Computed tomography (CT) scan showed a large mass with partial pleural adhesion which seemed to originate from the mediastinal pleura rather than the right middle lobe (Fig. 1a-b). The tumor was well-circumscribed measuring about 4 cm and displayed heterogeneous enhancement (parenchyma of the tumor showed moderate contrast enhancement; 21 HU (Hounsfield Unit) on pre-contrast image and 63 HU on post-contrast image) (Fig. 1c). On the lung window image, one discrete 11 mm nodule was also noted in the left lower lobe, raising the possibility of lung-to-lung metastases (Fig. 1d). Right pleural localized enclosing effusion and mild enlargement of mediastinal lymph nodes were additional radiological findings.

**Fig. 2:**
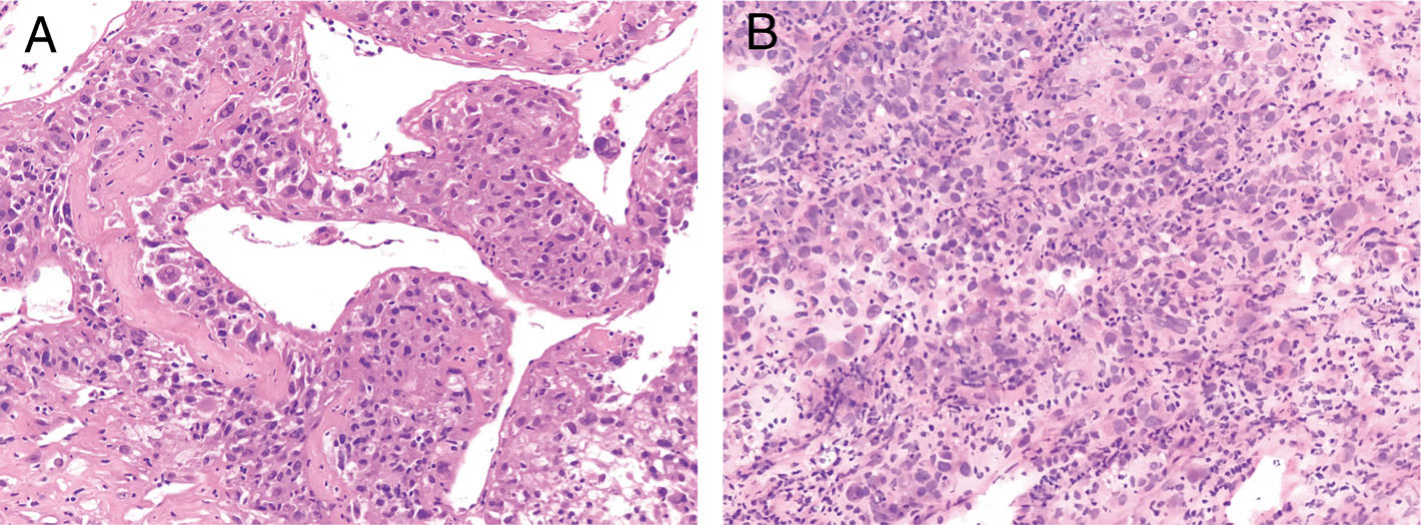
Intraoperative frozen section. **a** The middle lobe tumor composed of atypical epithelioid neoplasm cells organized in a angiopericytoma-like pattern, growing in the interstitial with abundant clear or granular cytoplasm. **b** The small solid nodule suggested a poor differentiated adenocarcinoma (original magnification × 200)

Surgical procedures were tentatively scheduled for tumor dissection of the middle lobe and wedge-resection of the left lower lobe. Intraoperative frozen section of the larger mass was interpreted as indeterminate for malignancy in view of the atypical tumor cells growing in the interstitial surrounding blood vessels while the small solid nodule was diagnosed as a poorly differentiated adenocarcinoma (Fig. 2). Based on the above analysis and the patient approval, supplementary lobectomy of the right middle lobe and lymph node dissection were performed subsequently.

**Fig. 3:**
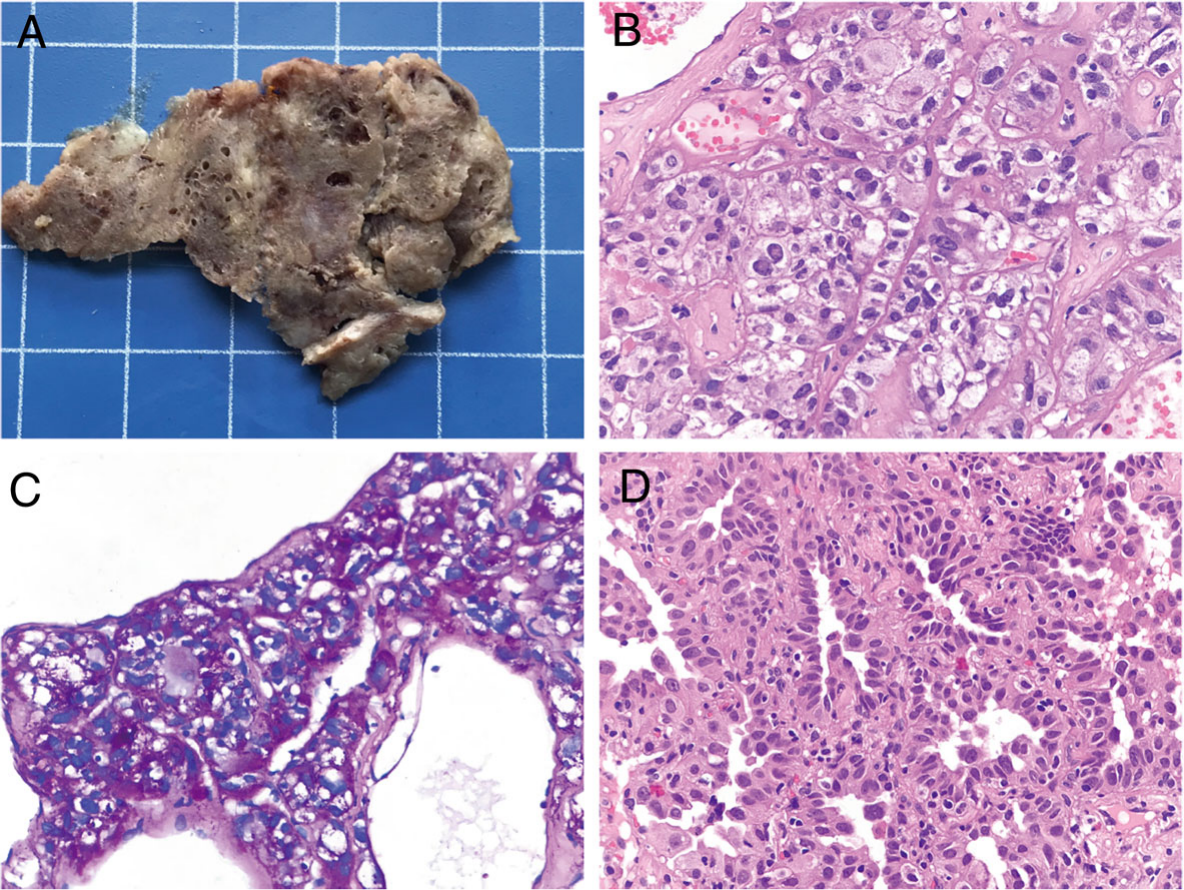
Gross and microscopic examination of the resected specimens. **a** Gross examination after fixation of the middle lobe mass had a grayish brown cut-surface and significant cystic lacunar structure. **b** Tumor parenchyma displayed perivascular haemangiopericytoma-like and solid sheets structure around sinusoidal blood vessels. **c** Periodic Acid-Schiff staining indicated abundant clear intracellular glycogen. **d** The small nodule of the left lower lobe was invasive lung adenocarcinoma presenting as acinar and solid subtype. **b**, **c** and **d**, original magnification × 400)

Gross specimen of the larger tumor showed a well-demarcated and non-encapsulated mass, with a grayish brown cut-surface and significant cystic lacunar structure (Fig. 3a). Microscopically, the tumor parenchyma was composed of epithelioid cells ranged in perivascular haemangiopericytoma-like patterns with clear or eosinophilic cytoplasm, with rich sinusoidal blood vessels (Fig. 3b). Trabecular-like and pellet-like growth pattern can be seen in some areas. Abundant clear intracellular glycogen displays positive Periodic Acid-Schiff staining with and without diastase digestion (Fig. 3c). Mass emergence of intra-nuclear pseudo-inclusions is an important morphological feature of this case. Neoplastic cells with obvious enlarged nucleoli and pathological mitosis were found. In addition, some dispersed bizarre hyperchromatic tumor giant cell (5/50 high-power fields) throughout the tumor is highly distinctive. The small solid nodular of the left lower lobe was confirmed as classic primary lung adenocarcinoma (Fig. 3d).

**Fig. 4:**
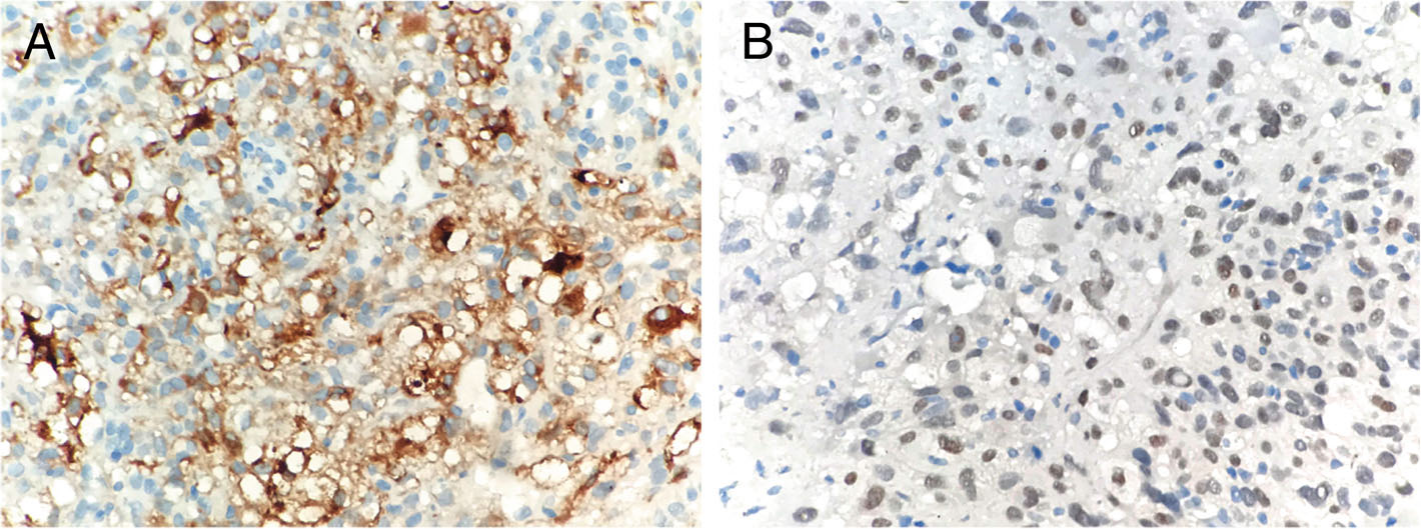
Immunohistochemistry staining of the malignant perivascular epithelioid cell tumor. **a** Tumor cells showed strong positivity for Melan-A, weak but diffuse and consistently positivity nuclear staining for TFE3 **b**

Immunohistochemistry showed strong positivity within tumor cells for Vimentin and Melan-A (Fig. 4a), weak but diffusely positive for TFE3 protein (Fig. 4b), while HMB45 was negative. The Ki-67 score was about 10%. The neoplastic cells failed to stain with epithelial marker pan-cytokeratin and epithelial membrane antigen (EMA), myogenic marker caldesmon, myogenin and α-smooth muscle actin (α-SMA), and additional antibodies including neuroendocrine markers.

TFE3 gene rearrangement was not identified by fluorescence in-situ hybridization and reverse transcription polymerase chain reaction (RT-PCR). The results of targeted molecular gene alteration including epidermal growth factor receptor (EGFR), anaplastic lymphoma kinase (ALK), ROS proto-oncogene 1(ROS1), kirsten rat sarcoma viral oncogene (K-ras) of these two tumors were all negative.

The patient underwent three courses of chemotherapy of combined paclitaxel (300 mg) and carboplatin (600 mg) after surgery. A follow up fluorine-18-fluorodeoxyglucose positron emission tomography/computed tomography (^18^F-FDG PET/CT) obtained 12 months after chemotherapy showed no metastatic lesions elsewhere. At present, the disease is stable and the patient is followed-up regularly.

## Discussion and conclusions

Conventional PEComas frequently harbor tuberous sclerosis complex (TSC) gene mutation [10]. A distinct subset of PEComa carrying transcription factor E3 (TFE3) gene rearrangements were identified arising in the uterine corpus, vagina, and pelvic and pulmonary tumor [11]. They typically are strongly positive for TFE3 and HMB45, with purely clear cell epithelioid cells and an alveolar architecture [12–14]. But it is still uncertain what the role of TFE3 is in malignant pulmonary PEComa. Recognition of this genetic abnormality may have critical therapeutic implications for aggressive tumor in future.

Including six cases in the English literature and two cases documented in the Chinese literature, a total of 8 aggressive or malignant PEComas of the lung have been reported [15–20]. These documents illustrated the invasive clinical behavior of the tumor from a series of angles, including tumor size, infiltrative growth pattern, nuclear grade, necrosis, and mitotic activity. According to most literatures, malignant PEComa of the lung also appeared radiologically as a solitary, well-defined nodule like benign clear cell sugar tumor, without cystic changes and calcification. Only one recently reported case showed spindle cell morphology and significant calcification.

Histopathologically, PEC tumor should be differentiated from primary clear cell pulmonary carcinoma, a variant of adenocarcinoma according to 2015 World Health Organization (WHO) classification of tumors of the lung, carcinoid, metastatic renal clear cell carcinoma, paraganglioma, primary intrapulmonary meningothelial neoplasm and malignant melanoma.

As with more and more recognized cases at numerous sites, diagnostic criteria for malignant PEComa continue to evolve. Firm diagnostic criteria for malignant pulmonary PEComa also need to be urgently established. Therefore, we put forward some suggestions for reference in diagnosis of malignant lung PEComa according to our experience and retrospective literature analysis. The primary diagnostic criteria include: prominent coagulative tumor necrosis; infiltrates and invade adjacent pleura or viscera; distant metastasis of homology; pathological mitotic figure≥1/50HP. Secondary diagnostic criteria are as follows: tumor size≥3 cm; spotty necrosis; high mitotic index≥5%; multinucleated tumor giant cell≥5/50HP; marked hyper-cellularity; nuclear atypia and pleomorphism; numerous intranuclear pseudoinclusion.

The criteria for the diagnosis of malignant lung PEComa that we recommend are that the tumor meets 1–2 major diagnostic criteria and/or one more secondary diagnostic criteria. If the tumor satisfies only 2 or more secondary diagnostic criteria, we suggest the diagnosis of lung PEComa with malignant potential.

Scope of surgical resection and lymph node dissection and subsequent medical treatment of malignant PEComa is another challenge. Complete surgical resection is probably the most effective treatment at present. As for the need for combined chemotherapy, it should be considered the degree of histologic differentiation and whether the tumor is associated with a primary malignant tumor. In our case, the middle lobe lesion was successfully removed from normal surrounding parenchyma and a wedge resection of left lower lobe was carried out. The right middle lobectomy and lymph node dissection were finally performed. Three courses of toxic chemotherapy were performed targeting for lung adenocarcinoma after operation, and we speculated that the prognosis of malignant PEComa may benefit from the strategy. Postoperatively, follow up ^18^F-FDG PET CT showed no evidence of residual tumor or metastatic lesions elsewhere.

Pulmonary malignant PEComa associated with a primary poorly differentiated adenocarcinoma has not been described previously. TFE3 gene mutation was not identified in this case. Due to this tumor’s uncommon occurrence, the diagnostic criteria of this entity are not widely known and may lead to misdiagnosis. Therefore, its malignant characteristics may be multifaceted and need to be addressed from different angles and more examples.

## Abbreviations

^18^F-FDG PET/CT: Fluorine-18-fluorodeoxyglucose positron emission tomography/computed tomography
ALK: Anaplastic lymphoma kinase
EGFR: Epidermal growth factor receptor
EMA: Epithelial membrane antigen
K-ras: Kirsten rat sarcoma viral oncogene
PEComa: Perivascular epithelioid cell tumor
ROS1: ROS proto-oncogene 1
TFE3: Transcription factor E3
TSC: Tuberous sclerosis complex
WHO: World Health Organization
α-SMA: α-smooth muscle actin
